# Terapia Antiplaquetária Dupla Combinada em Pacientes com Doença Arterial Coronariana em uma População na Turquia: DAPT-TR

**DOI:** 10.36660/abc.20240202

**Published:** 2024-11-07

**Authors:** Ahmet Öz, Kenan Toprak, Ertan Aydin, İbrahim Saraç, Mustafa Doğduş, Selçuk Opan, Mustafa Yenerçağ, Mustafa Begenc Tascanov, Ömer Kümet, Miraç Karaağaç, Murat Özmen, Bektaş Murat, Ömer Kertmen, Özkan Bekler, Sinan İnci, Mustafa Ahmet Huyut, Ahmet Özderya, Fahri Er, Mustafa Duran, İsa Ardahanlı, Mehmet Memduh Baş, Tuncay Güzel, Gökhan Ceyhun, İbrahim Halil Özdemir, Mehmet Burak Özen, Ramazan Gündüz, Aslan Erdoğan, İlyas Çetin, Veysel Özgür Barış, Çağrı Yayla, Medeni Karaduman, Lütfü Aşkın, Lütfü Bekar, Okan Tanrıverdi, Eyüp Özkan, Emrah Yeşil, Serhat Çalışkan, Zülfiye Kuzu, Berat Uğuz, Ferit Böyük, Ayşegül Ülgen Kunak, Selda Murat, Serkan Asil, Özkan Kayhan, Emrah Erdoğan, Ramazan Duz, Fahrettin Katkat, Tuba Ekin, Ersin İbişoğlu, Bilge Nazar Ateş, Burak Ayça, Asım Oktay Ergene, Mehdi Zoghi

**Affiliations:** 1 Department of Cardiology Health Science University Istanbul Training and Research Hospital Istanbul Turquia Department of Cardiology, Health Science University, Istanbul Training and Research Hospital, Istanbul - Turquia; 2 Harran University Faculty of Medicine Department of Cardiology Sanliurfa Turquia Harran University, Faculty of Medicine, Department of Cardiology, Sanliurfa - Turquia; 3 Giresun Training and Research Hospital Department of Cardiology Giresun Turquia Giresun Training and Research Hospital, Department of Cardiology, Giresun - Turquia; 4 Erzurum City Hospital Department of Cardiology Erzurum Turkey Erzurum City Hospital, Department of Cardiology, Erzurum - Turkey; 5 Uşak University Training and Research Hospital Department of Cardiology Usak Turquia Uşak University Training and Research Hospital, Department of Cardiology, Usak - Turquia; 6 Sanliurfa Training and Research Hospital Department of Cardiology Şanlıurfa Turquia Sanliurfa Training and Research Hospital, Department of Cardiology, Şanlıurfa - Turquia; 7 Samsun University Faculty of Medicine Department of Cardiology Samsun Turquia Samsun University, Faculty of Medicine, Department of Cardiology, Samsun - Turquia; 8 Van Training and Research Hospital Department of Cardiology Van Turquia Van Training and Research Hospital, Department of Cardiology, Van - Turquia; 9 İnönü University Turgut Özal Tıp Merkezi Training and Research Hospital Department of Cardiology Malatya Turquia İnönü University Turgut Özal Tıp Merkezi Training and Research Hospital, Department of Cardiology, Malatya - Turquia; 10 Eskişehir City Hospital Department of Cardiology Eskişehir Turquia Eskişehir City Hospital, Department of Cardiology, Eskişehir - Turquia; 11 Amasya University Sabuncuoğlu Şerefeddin Training and Research Hospital Department of Cardiology Amasya Turquia Amasya University Sabuncuoğlu Şerefeddin Training and Research Hospital, Department of Cardiology, Amasya - Turquia; 12 Mustafa Kemal University Faculty of Medicine Department of Cardiology Hatay Turquia Mustafa Kemal University, Faculty of Medicine, Department of Cardiology, Hatay - Turquia; 13 Aksaray University Faculty of Medicine Department of Cardiology Aksaray Turquia Aksaray University, Faculty of Medicine, Department of Cardiology, Aksaray - Turquia; 14 İstanbul Prof.Dr.Cemil Taşcıoğlu City Hospital Department of Cardiology İstanbul Turquia İstanbul Prof.Dr.Cemil Taşcıoğlu City Hospital, Department of Cardiology, İstanbul - Turquia; 15 Trabzon Kanuni Training and Research Hospital Department of Cardiology, Trabzon Turquia Trabzon Kanuni Training and Research Hospital, Department of Cardiology, Trabzon - Turquia; 16 Ağrı Training and Research Hospital Department of Cardiology, Ağrı Turquia Ağrı Training and Research Hospital, Department of Cardiology, Ağrı - Turquia; 17 Konya City Hospital Department of Cardiology Konya Turquia Konya City Hospital, Department of Cardiology, Konya - Turquia; 18 Bilcecik Şeyh Edebali University Faculty of Medicine Department of Cardiology Bilecik Turquia Bilcecik Şeyh Edebali University, Faculty of Medicine, Department of Cardiology, Bilecik - Turquia; 19 Private Meydan Hospital Department of Cardiology Şanlıurfa Turquia Private Meydan Hospital, Department of Cardiology, Şanlıurfa - Turquia; 20 Gazi Yaşargil Training and Research Hospital Department of Cardiology Diyarbakır Turquia Gazi Yaşargil Training and Research Hospital, Department of Cardiology, Diyarbakır - Turquia; 21 Atatürk University Faculty of Medicine Department of Cardiology Erzurum Turquia Atatürk University, Faculty of Medicine, Department of Cardiology, Erzurum - Turquia; 22 Manisa City Hospital Department of Cardiology Manisa Turquia Manisa City Hospital, Department of Cardiology, Manisa - Turquia; 23 Başakşehir Çam ve Sakura City Hospital Department of Cardiology İstanbul Turquia Başakşehir Çam ve Sakura City Hospital, Department of Cardiology, İstanbul - Turquia; 24 Gaziantep Dr. Ersin Arslan Training and Research Hospital Department of Cardiology Gaziantep Turquia Gaziantep Dr. Ersin Arslan Training and Research Hospital, Department of Cardiology, Gaziantep - Turquia; 25 Ankara Bilkent City Hospital Department of Cardiology Ankara Turquia Ankara Bilkent City Hospital, Department of Cardiology,Ankara – Turquia; 26 Van Yüzüncü Yıl University Faculty of Medicine Department of Cardiology Van Turquia Van Yüzüncü Yıl University, Faculty of Medicine, Department of Cardiology, Van - Turquia; 27 Gaziantep İslam Bilim ve Teknoloji University Faculty of Medicine Department of Cardiology Gaziantep Turquia Gaziantep İslam Bilim ve Teknoloji University, Faculty of Medicine, Department of Cardiology, Gaziantep - Turquia; 28 Hitit University Faculty of Medicine Department of Cardiology Çorum Turquia Hitit University, Faculty of Medicine, Department of Cardiology, Çorum - Turquia; 29 Adıyaman University Faculty of Medicine Department of Cardiology Adıyaman Turquia Adıyaman University, Faculty of Medicine, Department of Cardiology, Adıyaman – Turquia; 30 Mersin University Faculty of Medicine Department of Cardiology Mersin Turquia Mersin University, Faculty of Medicine, Department of Cardiology, Mersin - Turquia; 31 Bahçelievler State Hospital Department of Cardiology İstanbul Turquia Bahçelievler State Hospital, Department of Cardiology,İstanbul - Turquia; 32 Kayseri City Hospital Department of Cardiology Kayseri Turquia Kayseri City Hospital, Department of Cardiology, Kayseri - Turquia; 33 Bursa City Hospital Department of Cardiology Bursa Turquia Bursa City Hospital, Department of Cardiology, Bursa - Turquia; 34 Yedikule Chest Diseases and Thoracic Surgery Training and Research Hospital Department of Cardiology İstanbul Turquia Yedikule Chest Diseases and Thoracic Surgery Training and Research Hospital, Department of Cardiology, İstanbul - Turquia; 35 Bandırma Training and Research Hospital Department of Cardiology Balıkesir Turquia Bandırma Training and Research Hospital, Department of Cardiology, Balıkesir - Turquia; 36 Eskişehir Osmangazi University Faculty of Medicine Department of Cardiology Eskişehir Turquia Eskişehir Osmangazi University, Faculty of Medicine, Department of Cardiology, Eskişehir - Turquia; 37 Gülhane Training and Research Hospital Department of Cardiology Ankara Turquia Gülhane Training and Research Hospital, Department of Cardiology, Ankara - Turquia; 38 Kepez State Hospital Department of Cardiology Antalya Turquia Kepez State Hospital, Department of Cardiology, Antalya - Turquia; 39 Van Yüzüncü Yıl University Faculty of Medicine Department of Cardiology Van Turquia Van Yüzüncü Yıl University, Faculty of Medicine, Department of Cardiology, Van - Turquia; 40 İstanbul Training and Research Hospital Department of Cardiology İstanbul Turquia İstanbul Training and Research Hospital, Department of Cardiology, İstanbul - Turquia; 41 Kırşehir Ahi Evran University Training and Research Hospital Department of Cardiology Kırşehir Turquia Kırşehir Ahi Evran University Training and Research Hospital, Department of Cardiology, Kırşehir - Turquia; 42 Ankara University Faculty of Medicine Department of Cardiology Ankara Turquia Ankara University, Faculty of Medicine, Department of Cardiology, Ankara - Turquia; 43 Dokuz Eylul University Department of Cardiology Izmir Turquia Dokuz Eylul University, Department of Cardiology, Izmir - Turquia; 44 Ege University Department of Cardiology Izmir Turquia Ege University, Department of Cardiology, Izmir - Turquia

**Keywords:** Terapia Antiplaquetária Dupla, Hemorragia, Trombose

## Abstract

**Fundamento:**

A Terapia Antiplaquetária Dupla (DAPT, do inglês *dual antiplatelet therapy*) é o tratamento de escolha para pacientes com síndromes coronarianas agudas e crônicas, uma vez que ela reduz mortalidade e previne complicações trombóticas recorrentes. A avaliação da carga isquêmica e do risco de sangramento é crucial na escolha da DAPT e de sua duração.

**Objetivos:**

O objetivo de nosso estudo foi conduzir um acompanhamento clínico prospectivo de pacientes recebendo terapia combinada (AAS 75mg + clopidogrel 75 mg). Nosso estudo é um estudo multicêntrico, transversal, observacional, do tipo coorte.

**Métodos:**

Foram incluídos 1500 pacientes que iniciaram DAPT combinada para o tratamento de síndrome coronariana aguda ou síndrome coronariana crônica. Os desfechos primários foram internação hospitalar por qualquer motivo, internação por causa cardiovascular, infarto agudo do miocárdio, trombose de *stent*, revascularização de vaso alvo e sangramento; os desfechos secundários foram morte por qualquer razão ou por causa cardiovascular e acidente vascular cerebral. O nível de significância adotado na análise estatística foi 5%.

**Resultados:**

A idade mediana foi 63 anos; 78,5% dos pacientes estavam recebendo DAPT por síndrome coronariana aguda. As taxas de internação por causas cardiovasculares, de infarto agudo do miocárdio, de trombose de *stent* e de revascularização de vaso alvo foram 7,9%, 2,3%, 1,3% e 4,2%, respectivamente. Enquanto a taxa de sangramento segundo a escala BARC tipo 1 foi de 3,3%, a taxa de sangramento BARC tipo 5, 3, ou 2 foi 0,6%. As taxas dos desfechos secundários morte por qualquer razão, morte por causa cardiovascular e acidente vascular cerebral foram 0,5%, 0,3% e 0,3%, respectivamente.

**Conclusão:**

Nosso estudo mostrou que a DAPT em regime combinado de doses fixas de AAS e clopidogrel é eficaz e seguro em pacientes com síndrome coronariana aguda ou crônica adequadamente selecionados.

## Graphical abstract

**Central Illustration:**
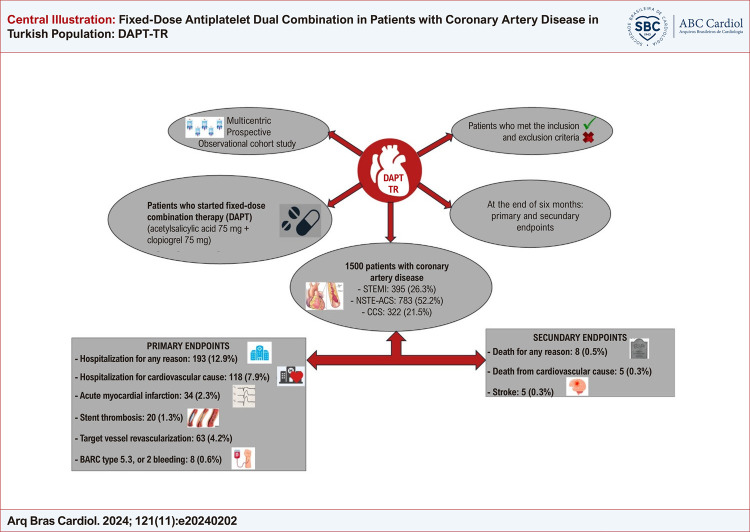
Fixed-Dose Antiplatelet Dual Combination in Patients with Coronary Artery Disease in Turkish Population: DAPT-TR

## Introdução

A doença isquêmica cardíaca é reconhecida como a causa mais comum de mortalidade em todo o mundo, e seu tratamento vem se tornado mais e mais importante dada à crescente incidência da doença. A Terapia Antiplaquetária Dupla (DAPT, do inglês *dual antiplatelet therapy*), que consiste em inibidores de P2Y12 (ticagrelor, prasugrel ou clopidogrel), combinados com ácido acetilsalicílico (AAS) é recomendada para reduzir mortalidade e mesmo prevenir complicações trombóticas recorrentes após eventos índices em pacientes submetidos à intervenção coronária percutânea (ICP) para síndromes coronarianas crônicas (SCC), em pacientes submetidos à ICP por infarto do miocárdio (IM) com Elevação do Segmento ST (IAMSST) e por Síndrome Coronariana Aguda (SCA) sem supradesnivelamento do segmento ST (SCA-SSST), ou em pacientes tratados clinicamente.^1,2^Nas diretrizes da ESC de 2017 para o manejo de pacientes com infarto agudo do miocárdio com elevação do segmento ST,^3^ DAPT com ticagrelor ou prasugrel (ou clopidogrel 75 mg/dia de dose de manutenção se o ticagrelor ou o prasugrel não estiver disponível ou for contraindicado) por 12 meses após a ICP em pacientes com baixo risco de sangramento em combinação com AAS 75-100mg/dia é uma recomendação classe 1, e DAPT por seis meses é recomendada com uma recomendação classe 2a em pacientes com um alto risco de sangramento.^3^Nas diretrizes de ESC de 2020 para o manejo de SCA sem elevação persistente do segmento ST,^4^ DAPT com ticagrelor ou prasugrel (ou clopidogrel 75 mg/dia de dose de manutenção se o ticagrelor ou o prasugrel não estiver disponível ou for contraindicado) mais AAS 75-100mg por 12 meses em pacientes submetidos à ICP e sem risco elevado de sangramento é recomendada com uma indicação classe 1. DAPT com ticagrelor ou clopidogrel 75 mg/dia de dose de manutenção é recomendada a pacientes acompanhados por tratamento clínico por qualquer razão e que não estejam em risco de sangramento excessivo.^4^Nas diretrizes da ESC de 2019 para o manejo de SCC, AAS 75-100 mg/dia com clopidogrel 75 mg/day por seis meses com indicação classe 1 é recomendado para pacientes submetidos à ICP após estratégia invasiva, independentemente do tipo de *stent*, em pacientes com alto risco de sangramento.^5^

A decisão sobre qual estratégia escolher na DAPT e a duração do tratamento baseia-se na avaliação da carga isquêmica e o risco de sangramento.^2^ Em pacientes tratados com AAS e clopidogrel em uma dose de 75 mg separadamente ou em dose fixa combinada, a combinação de doses fixas mostrou aumentar significativamente a adesão à medicação, embora nenhuma informação sobre eficácia ou segurança no seguimento foi apresentada.^6^ Assim, nosso objetivo é avaliar a segurança e a eficácia de uma terapia combinada de AAS (75 mg/dia) e clopidogrel (75 mg/dia) para doença arterial coronariana em um estudo prospectivo, observacional, multicêntrico.

### Métodos

O DAPT-TR é um estudo nacional, multicêntrico, prospectivo, observacional, do tipo coorte, conduzido em 37 universidades e dois hospitais privados em 27 cidades na Turquia. O protocolo do estudo foi revisado pelo comitê de ética do Hospital Istambul de treinamento e pesquisa, e aprovado em 10 de dezembro de 2021. De acordo com diretrizes locais e resultados de ensaios clínicos, 1500 pacientes que preencheram os critérios de inclusão e exclusão, que iniciaram o tratamento com DAPT combinada para doença arterial coronariana entre janeiro e dezembro de 2022, prescrito por um cardiologista, e estavam em tratamento por pelo menos um mês foram incluídos no estudo. Todos os pacientes com SCA receberam uma carga de 300mg de AAS combinado a 600mg de clopidogrel e pacientes com SCC receberam 300mg de clopidogrel e nenhum outro agente antiplaquetário. Todos os pacientes receberam uma combinação de DAPT em doses fixas como terapia de manutenção.

Critérios de inclusão:

- 18-90 anos de idade, de ambos os gêneros
- Indicação para DAPT por SCA e/ou doença arterial coronariana submetidos a tratamento intervencionista
- Pacientes em DAPT com dose fixa combinada há pelo menos um mês.

Critérios de exclusão

- Pacientes que não assinaram o termo de consentimento
- Pacientes sem indicação para DAPT por mais de um mês ou que não conseguiriam continuar o tratamento
- Cirurgia cardiovascular ou não-cardíaca planejada
- Doença hepática grave ou doença renal terminal
- Gestantes e lactantes
- Pacientes com uma expectativa de vida de menos de um ano
- Pacientes em tratamento para câncer
- Trombocitopenia persistente
- Pacientes em tratamento para anemia
- Pacientes com úlcera gastrointestinal
- Pacientes sabidamente resistentes à terapia antiplaquetária

Características basais, comorbidades, indicações para DAPT, medicamentos atuais, fração de ejeção ventricular esquerda no ecocardiograma, escores DAPT e PRECISE-DAPT foram registrados no momento da inclusão. Ao final do seguimento de cinco meses, os desfechos primários combinados – internação por qualquer motivo, internação por causa cardiovascular, infarto agudo do miocárdio, trombose do *stent*, revascularização do vaso alvo (RVA) e sangramento – e desfechos secundários – morte por qualquer razão, morte por causa cardiovascular e acidente vascular cerebral – foram registrados. Internações por causas cardiovasculares são definidas como insuficiência cardíaca, infarto agudo do miocárdio, trombose de *stent* e arritmias como fibrilação atrial ou taquicardia ventricular. Os critérios da Quarta Definição Universal do IM incluem um biomarcador cardíaco positivo e evidência clínica positiva, tais como sintomas de isquemia, alterações isquêmicas no eletrocardiograma, desenvolvimento de ondas Q patológicas e visualização de uma nova perda de miocárdio viável ou de anormalidade da motilidade da parede em uma região do miocárdio.^7^ Trombose de *stent* foi incluída nas diretrizes da *Academic Research Consortium* (ARC) sobre classificações de trombose de *stent* como eventos definitivos ou confirmados (sintomas sugestivos de SCA e confirmação angiográficas ou patológica de trombose de stent).^8^ A RVA foi definida como uma intervenção em qualquer artéria coronária tratada ou não tratada durante a ICP índice. Sangramento foi definido segundo os critérios do *Bleeding Academic Research Consortium* (BARC).^9^

### Análise estatística

As análises estatísticas foram conduzidas com o SPSS versão 21. As variáveis contínuas foram apresentadas em mediana e intervalo interquartil por não apresentarem distribuição normal. As variáveis categóricas foram expressas em frequências absolutas (n) e relativas (%). O teste de Kolmogorov-Smirnov foi usado para analisar normalidade da distribuição dos dados. O teste do qui-quadrado foi usado para comparar as variáveis categóricas entre os grupos. As variáveis contínuas sem distribuição normal foram comparadas pelo teste de Kruskal-Wallis. O teste post-hoc não foi utilizado por não exercer efeito sobre os desfechos clínicos, que foram o objetivo deste estudo. Um valor de p<0,05 foi considerado estatisticamente significativo, com um intervalo de confiança de 95%.

## Resultados

Foram incluídos 1500 pacientes que iniciaram DAPT combinada com doses fixas de medicamentos entre janeiro de 2022 e dezembro de 2022. Características demográficas, comorbidades e indicações para DAPT da população do estudo são descritas na Tabela 1. A idade mediana foi 63 (56-71) anos e 1006 (67,1%) pacientes eram do sexo masculino; 78,5% dos pacientes recebiam DAPT para SCA (26,3% IAMSST e 52,2% SCA-SSST). Idade, hipertensão, hiperlipidemia, história de tabagismo, CABG prévia e história de COVID foram estatisticamente diferentes entre os grupos. Valores medianos do escore PRECISE-DAPT foi 15 (8 – 23) e do escore DAPT foi 2 (1 – 3). Embora não tenha sido observada diferença significativa no escore PRECISE-DAPT entre os grupos, observou-se uma diferença significativa no escore DAPT. Os tratamentos medicamentosos concomitantes são listados na Tabela 2. A ocorrência de fração de ejeção ventricular esquerda inferior a 40% foi significativamente maior nos pacientes com IAMSST, assim como foi a utilização de ivabradina e antagonistas de mineralocorticoide nesse grupo. Os desfechos primários e secundários após seis meses de tratamento são apresentados na Tabela 3. Apesar das diferenças nas porcentagens nos desfechos primários e secundários, não foram observadas diferenças estatisticamente significativas entre os grupos. Os desfechos primários internação hospitalar por causas cardiovasculares, infarto agudo do miocárdio, trombose de *stent*, e RVA ocorreram em 7,9%, 2,3%, 1,3% e 4,2% dos pacientes, respectivamente. Sangramento tipo 5, 3, ou 2 (BARC) ocorreu em 3,3% dos pacientes, e BARC tipo 5, 3 ou 2 ocorreu em 0,6% dos pacientes. Os desfechos mortalidade por qualquer causa, mortalidade cardiovascular e acidente vascular cerebral ocorreram 0,5%, 0,3% e 0,3%, respectivamente (Figura Central).

**Tabela 1:** – Características demográficas, comorbidades e indicações de Terapia Antiplaquetária Dupla (DAPT) na população estudada

		Total n=1500	IAMSST n=395	SCA-SSST n=783	SCA n=322	Valor p
Indicação de DAPT, n (%)	IAMSST	395 (26,3%)				
	SCA-SSST	783 (52,2%)				
	SCA	322 (21,5%)				
Idade, anos		63 (56 – 71)	62 (54 – 71)	64 (56 – 72)	62 (56 – 69)	0,013
Sexo masculino, n (%)		1006 (67,1%)	277 (70,1%)	527 (67,3%)	202 (62,7%)	0,109
Diabetes mellitus, n (%)		543 (36,2%)	136 (34,4%)	277 (35,4%)	130 (40,4%)	0,203
Hipertensão, n (%)		948 (63,2%)	226 (57,2%)	492 (62,8%)	230 (71,4%)	<0,001
História familiar de DAC, n (%)		568 (37,9%)	151 (38,2%)	273 (34,9%)	144 (44,7%)	0,009
Hiperlipidemia, n (%)		831 (55,4%)	208 (52,7%)	423 (54%)	200 (62,1%)	0,022
CABG prévio, n (%)		130 (8,7%)	19 (4,8%)	85 (10,9%)	26 (8,1%)	0,002
Doença arterial periférica, n (%)		114 (7,6%)	26 (6,6%)	58 (7,4%)	30 (9,3%)	0,383
História de sangramento, n (%)		66 (4,4%)	12 (3%)	37 (4,7%)	17 (5,3%)	0,260
DPOC, n (%)		165 (11,0%)	54 (13,7%)	80 (10,2%)	31 (9,6%)	0,136
História de AVC/TIA, n (%)		60 (4,0%)	18 (4,6%)	35 (4,5%)	7 (2,2%)	0,130
IMC ≥ 30kg/m^2^, n (%)		301 (20,1%)	75 (19%)	165 (21,1%)	61 (18,9%)	0,596
História de tabagismo, n (%)	Não tabagista	619 (41,3%)	135 (34,2%)	331 (42,3%)	153 (47,5%)	<0,001
	Ex-tabagista	568 (37,9%)	152 (38,5%)	309 (39,5%)	107 (33,2%)	
	Tabagista atual	313 (20,9%)	108 (27,3%)	143 (18,3%)	62 (19,3%)	
Consumo de álcool, n (%)		167 (11,1%)	46 (11,6%)	97 (12,4%)	24 (7,4%)	0,056
História de COVID-19, n (%)		608 (40,5%)	160 (40,5%)	343 (43,8%)	105 (32,6%)	0,003

Variáveis categóricas (%) e variáveis contínuas apresentadas como mediana e intervalo interquartil; DAPT: terapia antiplaquetária dupla; DAC: doença arterial coronariana; CABG: bypass da artéria coronária; DPOC: doença pulmonar obstrutiva crônica; AIT: acidente isquêmico transitório; COVID-19: coronavirus disease 2019; IMC: índice de massa corporal; IAMSST: infarto do miocárdio (IM) com elevação do segmento ST; SCA: síndrome coronariana aguda; SCA-SSST: síndrome coronariana aguda sem supradesnivelamento do Segmento ST; AVC: acidente vascular cerebral.

**Tabela 2:** – Características clínicas e tratamentos medicamentosos concomitantes dos pacientes estudados

		Total n=1500	IAMSST n=395	SCA-SSST n=783	SCA n=322	Valor p
Pressão arterial sistólica, mm Hg		130 (120 – 142)	130 (119 – 140)	130 (120 – 145)	130 (119 – 140)	0,003
Pressão arterial diastólica, mm Hg		80 (70 – 85)	78 (70 – 85)	80 (71 – 88)	78 (70 – 85)	<0,001
Frequência cardíaca, pulso/min		75 (68 – 84)	75 (68 – 85)	77 (69 – 85)	74 (66 – 81)	0,001
Escore PRECISE-DAPT		15 (8 – 23)	13 (7 – 22)	15 (8 – 24)	15 (8 – 24)	0,128
Escore DAPT		2 (1 – 3)	2 (1 – 3)	2 (1 – 3)	2 (1 – 3)	<0,001
Hemoglobina, g/dL		13,7 (12,2 – 14,9)	14,0 (12,6 – 15,0)	13,6 (12,1 – 14,8)	13,6 (12,0 – 14,6)	0,005
LDL-colesterol, mg/dL		110 (85 – 137)	113 (89 – 138)	111 (86 – 138)	106 (77 – 135)	0,030
Fração de ejeção ventricular esquerda, n (%)	<%40	152 (10,1%)	53 (13,4%)	82 (10,5%)	17 (5,3%)	<0,001
	%40-49	340 (22,7%)	120 (30,4%)	162 (20,7%)	58 (18%)	
	≥%50	1008 (67,2%)	222 (56,2%)	539 (68,8%)	247 (76,7%)	
DCEI, n (%)		24 (1,6%)	3 (0,8%)	19 (2,4%)	2 (0,6%)	0,021
**Tratamentos medicamentosos concomitantes**						
Beta-bloqueador, n (%)		1310 (87,3%)	337 (85,3%)	688 (87,9%)	285 (88,5%)	0,358
IECA ou BRA, n (%)		1155 (77,0%)	316 (80%)	607 (77,5%)	232 (72%)	0,037
ARNI, n (%)		14 (0,9%)	2 (0,5%)	11 (1,4%)	1 (0,3%)	0,135
Inibidores de SGLT-2, n (%)		138 (9,2%)	42 (10,6%)	62 (7,9%)	34 (10,6%)	0,200
Estatinas de intensidade alta/moderada, n (%)		1244 (82,9%)	326 (82,5%)	650 (83,0%)	268 (83,2%)	0,966
Bloqueadores de canais de cálcio, n (%)		169 (11,3%)	39 (9,9%)	101 (18,3%)	29 (9%)	0,105
Nitratos, n (%)		245 (16,3%)	59 (14,9%)	143 (18,3%)	43 (13,4%)	0,091
Ranolazina, n (%)		225 (15,0%)	51 (12,9%)	132 (16,9%)	42 (13%)	0,109
Trimetazidina, n (%)		265 (17,7%)	60 (15,2%)	145 (18,5%)	60 (18,6%)	0,322
Ivabradina, n (%)		40 (2,7%)	19 (4,8%)	15 (1,9%)	6 (1,9%)	0,015
ARMs, n (%)		163 (10,9%)	61 (15,4%)	84 (10,7%)	18 (5,6%)	<0,001
IBP: n (%)		1174 (78,3%)	303 (76,7%)	607 (77,5%)	264 (82%)	0,179

Variáveis categóricas (%) e variáveis contínuas apresentadas principalmente em mediana e intervalos interquartis; LDL: lipoproteína de baixa densidade; DCEI: dispositivo cardíaco eletrônico implantável; DAPT: terapia antiplaquetária dupla; IECA: inibidores da enzima conversora de angiotensina; BRA: bloqueadores do recetor da angiotensina; ARNI: inibidor da neprilisina e do receptor de angiotensina; SGLT-2: cotransportador sódio-glicose 2; ARMs: antagonistas dos receptores de mineralocorticoides; IBP: inibidores de bomba de prótons.

**Tabela 3:** – Desfechos primários e secundários

	Total n=1500	IAMSST n=395	SCA-SSST n=783	SCA n=322	Valor p
**Desfechos primários**					
Internação hospitalar por qualquer motivo, n (%)	193 (12,9%)	53 (13,4%)	108 (13,8%)	32 (9,9%)	0,205
Internação por causa cardiovascular, n (%)	118 (7,9%)	31 (7,8%)	63 (8%)	24 (7,5%)	0,946
Infarto agudo do miocárdio, n (%)	34 (2,3%)	9 (2,3%)	20 (2,6%)	5 (1,6%)	0,573
Trombose de *stent*, n (%)	20 (1,3%)	9 (2,3%)	9 (1,2%)	2 (0,6%)	0,127
Revascularização de vaso alvo, n (%)	63 (4,2%)	21 (5,3%)	34 (4,3%)	8 (2,5%)	0,141
Sangramento BARC tipo 1, n (%)	50 (3,3%)	13 (3,3%)	25 (3,2%)	12 (3,7%)	0,905
Sangramento BARC tipo 5, 3 ou 2, n (%)	8 (0,6%)	1 (0,3%)	5 (0,6%)	2 (0,6%)	0,672
**Desfechos secundários**					
Morte por qualquer razão, n (%)	8 (0,5%)	3 (0,8%)	3 (0,4%)	2 (0,6%)	0,684
Morte por causa cardiovascular, n (%)	5 (0,3%)	2 (0,5%)	2 (0,3%)	1 (0,3%)	0,777
Acidente vascular cerebral, n (%)	5 (0,3%)	2 (0,5%)	3 (0,4%)	0	0,474

BARC: Bleeding Academic Research Consortium; IAMSST: infarto do miocárdio com elevação do segmento ST; SCA: síndrome coronariana aguda; SCA-SSST: síndrome coronariana aguda sem supradesnivelamento do segmento ST.

## Discussão

Em pacientes submetidos a tratamento clínico ou ICP por IAMSST, SCA-SSST ou SCC, as diretrizes atuais mostraram que a adição de inibidores de P2Y12 (ticagrelor, prasugrel ou clopidogrel) ao AAS durante períodos apropriados tem efeitos favoráveis sobre a mortalidade.^3-5^ A combinação da carga isquêmica com o risco de sangramento é crucial na escolha do tipo e da duração da DAPT. Idade avançada, presença de SCA, diabetes mellitus, doença renal crônica, características angiográficas e escore de risco isquêmico elevado (SYNTAX 2, GRACE, TIMI, DAPT) devem ser considerados na avaliação da carga isquêmica. Na avaliação de risco de sangramento, devem ser considerados história de sangramento maior, história de acidente vascular cerebral, anemia, diminuição na contagem de plaquetas, doenças malignas, idade avançada, doença hepática grave em uso de anticoagulantes, e elevado escore de risco de sangramento (ARC-HBR, PRECISE-DAPT). A DAPT é prescrita considerando as recomendações das diretrizes atuais, de modo a equilibrar a carga isquêmica, a qual é um indicador de eficácia, e o risco de sangramento, que é um indicador de segurança. A terapia combinada com doses fixas de AAS (75mg) e clopidogrel (75mg) foi efetiva e segura em pacientes elegíveis com SCC ou aguda. No estudo CURE, que observou os efeitos da dose de AAS quando usado sozinho ou em combinação com clopidogrel em pacientes com SCA, doses mais altas de AAS (>100mg) levaram a taxas mais altas de sangramento e ausência de eficácia adicional.^10^ Na doença cardiovascular aterosclerótica, dados atuais recomendam uma dose diária de AAS entre 75 e 100mg. Na França, em um estudo com 380 pacientes, AAS 75mg e clopidogrel, administrados separadamente ou de maneira combinada, melhoraram a adesão medicamentosa do paciente, mas não foram avaliados em termos de eficácia e segurança.^6^ Nosso estudo é o primeiro estudo prospectivo, multicêntrico, observacional a investigar a segurança e a eficácia da combinação de doses fixas de AAS (75mg) e clopidogrel (75mg) nesses pacientes.

Em relação aos desfechos primários, a taxa de internação por causas cardiovasculares incluindo insuficiência cardíaca, infarto agudo do miocárdio, trombose de *stent* e arritmias como fibrilação atrial ou taquicardia ventricular foi de 7,9%. Cronologicamente, o uso de AAS isolado, seguido de DAPT com AAS e clopidogrel, e finalmente AAS com ticagrelor e/ou prasugrel, inibidores mais potentes do receptor para adenosina difosfato P2Y12, mostrou reduzir o risco de eventos isquêmicos no miocárdio em pacientes com SCA.^2^ Estudos prospectivos envolvendo pacientes com SCA ou pacientes submetidos à ICP em que a terapia com clopidogrel foi usada mostrou diferenças em termos de desfechos primários. Quando olhamos para os estudos conduzidos com pacientes com SCA, no estudo TRITON-TIMI 38,^11^ IM foi observado em 9,7% dos pacientes no grupo recebendo clopidogrel.^11^ No estudo CLARITY-TIMI 28,^12^ a taxa de IM recorrente no braço clopidogrel foi 2,5%,^12^ e no estudo CURE,^13^ a taxa de IM foi de 5,2% em 6,259 pacientes usando clopidogrel.^13^ No estudo CURRENT-OASIS 7,^14^ a taxa de IM foi de 2% no grupo recebendo dose dupla e 2,6% no grupo recebendo dose padrão,^14^ e no estudo PLATO, a taxa de IM foi 6,9% no braço clopidogrel.^15^ Assim, como se pode observar, apesar da taxa de IM variar entre os estudos de SCA usando clopidogrel, em nosso estudo, essa taxa foi baixa – 2,3% na população total, 2,3% no grupo IAMSST e 2,6% no grupo SCA-SSST. Quanto aos estudos conduzidos com paciente com SCC, no estudo EXCELLENT,^16^ IM foi observado em 1,8% dos 722 pacientes no grupo de tratamento de seis meses de duração.^16^ No estudo PRODIGY,^17^ a taxa de IM foi 4,2% no grupo submetido à DAPT de curta duração (seis meses), e no grupo CREDO,^18^ IM ocorreu em 6,6% dos pacientes no braço clopidogrel de 1053 pacientes.^18^ Em uma meta-análise de 10 ensaios controlados randomizados sobre a duração ótima da DAPT após ICP com *stents* farmacológicos, a taxa de IM foi de 1,6% no tratamento de curta duração.^19^ Portanto, embora a taxa de IM varie em vários estudos usando clopidogrel, essa taxa foi baixa (como 1,6%) no subgrupo de pacientes com SCC.

Em um estudo multicêntrico, prospectivo, Oh et al.^20^ investigaram o potencial de inibição plaquetária de uma dose fixa de AAS com clopidogrel combinados e compararam com doses separadas em pacientes submetidos à ICP com *stent* farmacológico. Os autores demonstraram que a eficácia da inibição plaquetária terapia combinada com doses fixas das medicações não foi diferente que a da terapia com doses separadas de AAS e clopidogrel, sem ocorrência de eventos cardiovasculares sérios.^20^ Ainda, em um estudo randomizado, Choi et al.^21^ mostraram que as características farmacocinéticas da combinação de AAS e clopídogrel foram bioequivalentes a dos medicamentos administrados separadamente. Essas características farmacocinéticas podem explicar a eficácia da terapia combinada nos desfechos clínicos em comparação à administração das medicações de maneira isolada.^21^

Quando olhamos para trombose de *stent*, o qual é avaliado como outro indicador de eficácia, diferentes taxas têm sido relatadas entre os estudos, principalmente devido a variações na definição de trombose de *stent* e seleção dos pacientes. No estudo PLATO,^15^a trombose de *stent* ocorreu em 1,9% no braço clopidogrel. No estudo TRITON-TIMI 38,^11^ a taxa de trombose definitiva ou provável no grupo clopidogrel foi 2,4%. No ensaio CIRRENT-OASIS 7,^14^ observou-se uma taxa de trombose de *stent* definitiva de 0,7% no grupo recebendo dose dupla que 1,3% no grupo recebendo dose padrão.^14^ Embora a taxa de trombose de *stent* varie entre estudos com pacientes com SCA, em nosso estudo, ela ocorreu em 1,3% dos pacientes – 2,3% no subgrupo IAMSST e 1,3% no subgrupo SCA-SSST. Nos estudos EXCELLENT^16^ e PRODIGY,^17^ trombose de *stent* definitiva e provável nos pacientes em DAPT por seis meses foram de 0,9% e 1,5%, respectivamente. Em uma meta-análise de 10 estudos usando clopidogrel após *stents* farmacológicos em pacientes com SCC, a taxa de trombose de *stent* foi de 0,52%.^19^ Em nosso estudo, a taxa de trombose de *stent* definitiva foi de 0,6% no subgrupo com SCC. Enquanto a taxa de RVA de emergência foi de 3,7% no estudo TRITON-TIMI,^11^a taxa de revascularização após randomização foi de 11,5% no estudo CURE,^13^ a taxa de RVA foi 13,1% no estudo CREDO.^18^Em nosso estudo, a taxa de RVA foi de 4,2%. Tais diferenças significativas entre os estudos devem-se provavelmente a vários fatores, incluindo estrutura do *stent*, comorbidades, e período de seguimento.

Quanto ao desfecho secundário, a taxa de acidente vascular cerebral foi de 0,3% em nosso estudo. Nos estudos CHARISMA,^22^ PLATO,^15^ CURE^13^ e CREDO,^18^ as taxas de acidente vascular cerebral foram 1,9%, 1,3%, 1,2% e 0,9%, respectivamente. Acreditamos que tais variações devem-se às diferentes características das populações dos estudos.

Sangramento é usado para avaliação de desfecho de segurança das terapias antiplaquetária, antiagregante e anticoagulante. Várias classificações de sangramento foram estudadas, incluindo o TIMI (*Thrombolysis in Myocardial Infarction*), BARC, REPLACE-2 e GUSTO.^9^ No estudo CLARITY-TIMI,^12^ a taxa de sangramento maior segundo o escore TIMI foi de 1,9% e a de sangramento menor de 1,3% no grupo submetido à ICP.^12^ No estudo TRITON-TIMI 38, sangramento maior (TIMI) não relacionado à CABG ocorreu em 1,8% dos pacientes tratados com clopidogrel.^11^ No estudo CURRENT-OASIS 7,^14^ a taxa de sangramento maior e grave segundo definição CURRENT foi de 2,7% no grupo que recebeu dose dupla e 1,9¨no grupo que recebeu dose padrão. No estudo EXCELLENT,^17^ a taxa de sangramento maior (escore TIMI) foi de 0,3% no grupo em DAPT de curta duração.^16^ No estudo PRODIGY,^17^ a taxa de BARC tipo 5, 3, ou 2 foi de 3,5% e a de sangramento maior (escore TIMI) foi 0,6% no grupo em DAPT por seis meses. No estudo CREDO,^18^a taxa e sangramento maior (escore TIMI) não relacionado ao procedimento foi de 1,2% no grupo de pacientes tratados com clopidogrel. Em nosso estudo, utilizamos a classificação de BARC, e a taxa de BARC tipo 1 foi de 3,3%, de BARC tipo 5, 3 ou 2 foi de 0,6%; no subgrupo IAMSST, a taxa de BARC tipo 1 foi de 3,3%, de BARC tipo 5, 3 ou 2 foi de 0,3%; no subgrupo SCA-SSST, a taxa de BARC tipo 1 foi de 3,2%, de BARC tipo 5, 3 ou 2 foi de 0,6%; e no subgrupo SCC, a taxa de BARC tipo 1 foi de 3,7%, de BARC tipo 5, 3 ou 2 foi de 0,6%. Isso sugere que as diferenças entre os estudos podem ser atribuídas aos critérios de inclusão e de exclusão. Jung et al.^23^ avaliaram o perfil de segurança da terapia combinada com doses fixas de medicamentos em um ensaio randomizado. Não foram observados eventos adversos ou morte na população do estudo.

### Limitações do estudo

A primeira limitação do nosso estudo é a ausência de estudos comparando outras DAPTs ou tratamentos com uso separado de AAS e clopidogrel. A segunda é a inclusão de doenças que impõem um alto risco de sangramento nos critérios de exclusão do paciente.

## Conclusões

Nosso estudo demonstra que o uso combinado de doses fixas de medicamentos na DAPT é eficaz e seguro em pacientes com SCA ou SCC, selecionados conforme a carga isquêmica e o risco de sangramento.
