# Supplementary material for: Terapia Antiplaquetária Dupla Combinada em Pacientes com Doença Arterial Coronariana em uma População na Turquia: DAPT-TR
Source: Arq Bras Cardiol. 2024 Nov 7;121(11):e20240202. [Article in Portuguese] doi: 10.36660/abc.20240202 (PMC11634293; doi:10.36660/abc.20240202)
Supplement: Translation

# Translation: Fixed-Dose Antiplatelet Dual Combination in Patients with Coronary Artery Disease in Turkish Population: DAPT-TR

**DOI:** 10.36660/abc.20240202i

**Authors:** Ahmet Öz, Kenan Toprak, Ertan Aydin, İbrahim Saraç, Mustafa Doğduş, Selçuk Opan, Mustafa Yenerçağ, Mustafa Begenc Tascanov, Ömer Kümet, Miraç Karaağaç, Murat Özmen, Bektaş Murat, Ömer Kertmen, Özkan Bekler, Sinan İnci, Mustafa Ahmet Huyut, Ahmet Özderya, Fahri Er, Mustafa Duran, İsa Ardahanlı, Mehmet Memduh Baş, Tuncay Güzel, Gökhan Ceyhun, İbrahim Halil Özdemir, Mehmet Burak Özen, Ramazan Gündüz, Aslan Erdoğan, İlyas Çetin, Veysel Özgür Barış, Çağrı Yayla, Medeni Karaduman, Lütfü Aşkın, Lütfü Bekar, Okan Tanrıverdi, Eyüp Özkan, Emrah Yeşil, Serhat Çalışkan, Zülfiye Kuzu, Berat Uğuz, Ferit Böyük, Ayşegül Ülgen Kunak, Selda Murat, Serkan Asil, Özkan Kayhan, Emrah Erdoğan, Ramazan Duz, Fahrettin Katkat, Tuba Ekin, Ersin İbişoğlu, Bilge Nazar Ateş, Burak Ayça, Asım Oktay Ergene, Mehdi Zoghi

**Affiliations:** 1 Turkey Department of Cardiology, Health Science University, Istanbul Training and Research Hospital, Istanbul – Turkey; 2 Turkey Harran University, Faculty of Medicine, Department of Cardiology, Sanliurfa – Turkey; 3 Turkey Giresun Training and Research Hospital, Department of Cardiology, Giresun – Turkey; 4 Turkey Erzurum City Hospital, Department of Cardiology, Erzurum – Turkey; 5 Turkey Uşak University Training and Research Hospital, Department of Cardiology, Usak – Turkey; 6 Turkey Sanliurfa Training and Research Hospital, Department of Cardiology, Şanlıurfa – Turkey; 7 Turkey Samsun University, Faculty of Medicine, Department of Cardiology, Samsun – Turkey; 8 Turkey Van Training and Research Hospital, Department of Cardiology, Van – Turkey; 9 Turkey İnönü University Turgut Özal Tıp Merkezi Training and Research Hospital, Department of Cardiology, Malatya – Turkey; 10 Turkey Eskişehir City Hospital, Department of Cardiology, Eskişehir – Turkey; 11 Turkey Amasya University Sabuncuoğlu Şerefeddin Training and Research Hospital, Department of Cardiology, Amasya – Turkey; 12 Turkey Mustafa Kemal University, Faculty of Medicine, Department of Cardiology, Hatay – Turkey; 13 Turkey Aksaray University, Faculty of Medicine, Department of Cardiology, Aksaray – Turkey; 14 Turkey İstanbul Prof.Dr.Cemil Taşcıoğlu City Hospital, Department of Cardiology, İstanbul – Turkey; 15 Turkey Trabzon Kanuni Training and Research Hospital, Department of Cardiology,Trabzon – Turkey; 16 Turkey Ağrı Training and Research Hospital, Department of Cardiology, Ağrı – Turkey; 17 Turkey Konya City Hospital, Department of Cardiology, Konya – Turkey; 18 Turkey Bilcecik Şeyh Edebali University, Faculty of Medicine, Department of Cardiology, Bilecik – Turkey; 19 Turkey Private Meydan Hospital, Department of Cardiology, Şanlıurfa – Turkey; 20 Turkey Gazi Yaşargil Training and Research Hospital, Department of Cardiology, Diyarbakır – Turkey; 21 Turkey Atatürk University, Faculty of Medicine, Department of Cardiology, Erzurum – Turkey; 22 Turkey Manisa City Hospital, Department of Cardiology, Manisa – Turkey; 23 Turkey Başakşehir Çam ve Sakura City Hospital, Department of Cardiology, İstanbul – Turkey; 24 Turkey Gaziantep Dr. Ersin Arslan Training and Research Hospital, Department of Cardiology, Gaziantep – Turkey; 25 Turkey Ankara Bilkent City Hospital, Department of Cardiology, Ankara – Turkey; 26 Turkey Van Yüzüncü Yıl University, Faculty of Medicine, Department of Cardiology, Van – Turkey; 27 Turkey Gaziantep İslam Bilim ve Teknoloji University, Faculty of Medicine, Department of Cardiology, Gaziantep – Turkey; 28 Turkey Hitit University, Faculty of Medicine, Department of Cardiology, Çorum – Turkey; 29 Turkey Adıyaman University, Faculty of Medicine, Department of Cardiology, Adıyaman – Turkey; 30 Turkey Mersin University, Faculty of Medicine, Department of Cardiology, Mersin – Turkey; 31 Turkey Bahçelievler State Hospital, Department of Cardiology, İstanbul – Turkey; 32 Turkey Kayseri City Hospital, Department of Cardiology, Kayseri – Turkey; 33 Turkey Bursa City Hospital, Department of Cardiology, Bursa – Turkey; 34 Turkey Yedikule Chest Diseases and Thoracic Surgery Training and Research Hospital, Department of Cardiology, İstanbul – Turkey; 35 Turkey Bandırma Training and Research Hospital, Department of Cardiology, Balıkesir – Turkey; 36 Turkey Eskişehir Osmangazi University, Faculty of Medicine, Department of Cardiology, Eskişehir – Turkey; 37 Turkey Gülhane Training and Research Hospital, Department of Cardiology, Ankara – Turkey; 38 Turkey Kepez State Hospital, Department of Cardiology, Antalya – Turkey; 39 Turkey Van Yüzüncü Yıl University, Faculty of Medicine, Department of Cardiology, Van – Turkey; 40 Turkey İstanbul Training and Research Hospital, Department of Cardiology, İstanbul – Turkey; 41 Turkey Kırşehir Ahi Evran University Training and Research Hospital, Department of Cardiology, Kırşehir – Turkey; 42 Turkey Ankara University, Faculty of Medicine, Department of Cardiology, Ankara – Turkey; 43 Turkey Dokuz Eylul University, Department of Cardiology, Izmir – Turkey; 44 Turkey Ege University, Department of Cardiology, Izmir – Turkey

**Keywords:** Dual Anti-Platelet Therapy, Hemorrhage, Thrombosis

## Abstract

**Background:**

Dual antiplatelet therapy (DAPT) is the treatment of choice for patients with acute and chronic coronary syndromes as it reduces mortality and prevents recurrent thrombotic complications. The assessment of both ischaemic burden and bleeding risk is crucial in deciding which DAPT to choose and how long it should be continued.

**Objectives:**

The aim of our study was to perform prospective clinical follow-up of patients receiving fixed-dose combination therapy (ASA 75 mg + clopidogrel 75 mg). Our study is a multicentric, cross-sectional, observational, cohort study.

**Methods:**

A total of 1500 patients who were started on fixed-dose combination DAPT for acute or chronic coronary syndrome were included in the study. Primary endpoints were hospitalization for any reason, hospitalization for cardiovascular cause, acute myocardial infarction, stent thrombosis, target vessel revascularization and bleeding; the secondary endpoints were death for any reason or cardiovascular cause and stroke. The significance level adopted in the statistical analysis was 5%.

**Results:**

Median age was 63 years; 78.5% of the patients were receiving DAPT treatment for acute coronary syndrome. The rates of hospitalization for cardiovascular reasons, acute myocardial infartion, stent thrombosis and target-vessel revascularization were 7.9%, 2.3%, 1.3% and 4.2%, respectively. While the rate of BARC type 1 bleeding was 3.3%, the rate of BARC type 5, 3, or 2 bleeding was 0.6%. The secondary endpoints which were death from any cause, cardiovascular death and stroke were 0.5%, 0.3% and 0.3%, respectively. Conclusion: Our study shows that fixed-dose combination therapy is effective and safe in appropriately selected patients with acute or chronic coronary syndromes.

## Introduction

Ischemic heart disease is recognised as the most common cause of death worldwide and its treatment management has become more and more important due to the increasing incidence of the disease. Dual antiplatelet therapy (DAPT) consisting of P2Y12 inhibitors (ticagrelor, prasugrel or clopidogrel) combined with acetylsalicylic acid (ASA) is recommended to reduce mortality and prevent recurrent thrombotic complications after index events in patients undergoing percutaneous coronary intervention (PCI) for chronic coronary syndromes (CCS), in patients undergoing PCI for ST-elevation myocardial infarction (STEMI) and non-ST-segment elevation acute coronary syndrome (NSTE-ACS), or in patients treated medically.^1,2^ In the 2017 ESC Guideline for Acute Myocardial Infarction (MI) in Patients Presenting with ST-Segment Elevation,^3^ DAPT with ticagrelor or prasugrel (or clopidogrel 75 mg/day maintenance dose if ticagrelor or prasugrel is not available or contraindicated) for 12 months after PCI in patients with low bleeding risk in combination with ASA 75-100 mg/day is a class 1 recommendation, while DAPT for six months is a class 2a recommendation in patients with high bleeding risk.^3^ Similarly, in the 2020 ESC guidelines for ACS without persistent ST-segment elevation,^4^ DAPT with ticagrelor or prasugrel (or clopidogrel 75 mg/day maintenance dose if ticagrelor or prasugrel is not available or contraindicated) plus ASA 75-100 mg/day for 12 months in patients undergoing PCI and not at high risk of bleeding is recommended with a class 1 indication. DAPT with ticagrelor or clopidogrel 75 mg/day maintenance dose is recommended in patients who are followed up with medical treatment for any reason and who are not at risk of excessive bleeding.^4^ In the 2019 ESC guidelines for chronic coronary syndromes, ASA 75-100 mg/day with clopidogrel 75 mg/day for 6 months with class 1 indication is recommended for patients undergoing PCI after invasive strategy, regardless of stent type, in patients without high bleeding risk.^5^

The decision on which strategy to choose in DAPT and the duration of treatment is based on the evaluation of ischaemic burden and bleeding risk.^2^ In patients treated with ASA and clopidogrel at a dose of 75 mg separately or in fixed-dose combination, fixed-dose combination has been shown to significantly increase drug adherence, but no information on efficacy or safety in follow-up has been provided.^6^ Therefore, we aimed to evaluate the safety and efficacy of a fixed-dose ASA (75 mg/day) – clopidogrel (75mg/day) combination for coronary artery disease in a prospective, multicentric, observational study.

## Methods

DAPT-TR is a national, multicentric, prospective and observational cohort study conducted in 37 universities and two private hospitals in 27 cities in Turkey. The study protocol was reviewed by the Ethics Committee of Istanbul Training and Research Hospital and approved on 10 December 2021. In accordance with current guidelines and results of clinical trials, 1500 patients who met the inclusion and exclusion criteria, who were started on fixed dose combination DAPT treatment for coronary artery disease between January 2022 and December 2022 by a cardiologist and received this drug treatment for at least 1 month, were included in the study. All patients with acute coronary syndrome were loaded with 300 mg ASA along with 600 mg clopidogrel and patients with CCS received 300 mg clopidogrel and no other antiplatelet agents were used. All patients received fixed-dose combination DAPT as maintenance therapy.

Inclusion criteria:

- 18-90 years of age, regardless of gender
- Indication for DAPT due to acute coronary syndrome and/or coronary artery disease undergoing interventional treatment
- Patients who have been on fixed-dose combination therapy for at least one month

Exclusion criteria:

- Patients who did not sign a consent form
- Those who had no indication for dual antiplatelet therapy for more than one month or who was not be able to continue treatment
- Non-cardiac or cardiovascular surgery planned
- Severe liver disease or end-stage renal failure
- Pregnant and breastfeeding women
- Patients with a life expectancy of less than one year
- Those undergoing cancer treatment
- Persistent thrombocytopenia
- Those receiving treatment for anaemia
- Known gastrointestinal ulcers
- Patients known to be resistant to antiplatelet therapy

Baseline characteristics, comorbidities, indications for DAPT, current medication, echocardiographic left ventricular ejection fraction, DAPT and PRECISE-DAPT scores were recorded at the time of inclusion. At the end of five-month follow-up, the combined primary endpoints – hospitalization for any reason, hospitalization for cardiovascular cause, acute MI, stent thrombosis, target vessel revascularization (TVR) and bleeding] and secondary endpoints (death for any reason, death from cardiovascular cause, stroke) were recorded. Hospitalizations for cardiovascular reasons are defined as heart failure, acute MI, stent thrombosis and rhythm disorders such as atrial fibrillation or ventricular tachycardia. The criteria according to the Fourth Universal Definition of MI include a positive cardiac biomarker and corroborating clinical evidence, such as ischaemic symptoms, new ischaemic electrocardiographic changes, development of pathological Q waves and visualization of a new loss of viable myocardium or regional wall motion abnormality.^7^ Stent thrombosis was included in the 2008 Academic Research Consortium (ARC) guidelines on stent thrombosis classifications as definite or confirmed events (symptoms suggestive of acute coronary syndrome and angiographic or pathological confirmation of stent thrombosis).^8^ TVR was defined as an intervention in any coronary artery treated or untreated during the index PCI. Bleeding was defined according to the Bleeding Academic Research Consortium (BARC) criteria.^9^

### Statistical analysis

Statistical analyses were carried out with SPSS version 21. Continuous variables were presented as median and interquartile range as they did not show normal distribution. Categorical variables were expressed as absolute (n) and relative frequencies (%). The Kolmogorov-Smirnov test was used to assess the normality of data distribution. Chi-square test was used to compare categorical variables between groups. Continuous variables without normal distribution were compared using the Kruskal-Wallis test, and post hoc test was not used because it had no effect on the clinical outcomes, which was the aim of the study. A p<0.05 was considered significant with 95% confidence interval.

## Results

A total of 1,500 patients who started fixed-dose combination therapy between January 2022 and December 2022 were included. Demographic characteristics, comorbid conditions and DAPT indications of the study population are shown in Table 1. Median age was 63 (56 – 71) years and 1006 (67.1%) patients were male; 78.5% of the patients were receiving DAPT treatment for acute coronary syndrome (26.3% STEMI and 52.2% NSTE-ACS). Age, hypertension, hyperlipidemia, smoking history, previous CABG and COVID history were found to be statistically significant between the groups. The median PRECISE-DAPT score was 15 (8 – 23) and the median DAPT score was 2 (1 – 3). Although there was no significant difference between the groups in the PRECISE-DAPT score, a significant difference was observed in the DAPT score. The concurrent drug treatments are listed in Table 2. Occurence of left ventricular ejection fraction <40% was found to be significantly higher in the STEMI group, and accordingly, the use of ivabradine and mineralocorticoid receptor antagonists was found to be statistically higher. The primary and secondary endpoints after six months of treatment, are shown in Table 3. Although there were percentage differences in the primary and secondary endpoints, no statistically significant difference was found between the groups. The primary endpoints consisting of hospitalization for cardiovascular reasons, acute MI, stent thrombosis and TVR were 7.9%, 2.3%, 1.3% and 4.2%, respectively. BARC type 1 bleeding occurred in 3.3% of patients, while BARC type 5, 3, or 2 bleeding occurred in 0.6%. The secondary endpoints consisting of death from any cause, cardiovascular death and stroke were 0.5%, 0.3% and 0.3%, respectively (Central Illustration).

**Table 1:** – Demographic characteristics, comorbid conditions and dual antiplatelet therapy (DAPT) indications of the study population

		Total n=1500	STEMI n=395	NSTE-ACS n=783	CCS n=322	p value
DAPT indications, n (%)	STEMI	395 (26.3%)				
	NSTE-ACS	783 (52.2%)				
	CCS	322 (21.5%)				
Age, years		63 (56 – 71)	62 (54 – 71)	64 (56 – 72)	62 (56 – 69)	0.013
Gender (male), n (%)		1006 (67.1%)	277 (70.1%)	527 (67.3%)	202 (62.7%)	0.109
Diabetes mellitus, n (%)		543 (36.2%)	136 (34.4%)	277 (35.4%)	130 (40.4%)	0.203
Hypertension, n (%)		948 (63.2%)	226 (57.2%)	492 (62.8%)	230 (71.4%)	<0.001
Family history of CAD, n (%)		568 (37.9%)	151 (38.2%)	273 (34.9%)	144 (44.7%)	0.009
Hyperlipidemia, n (%)		831 (55.4%)	208 (52.7%)	423 (54%)	200 (62.1%)	0.022
Prior CABG,n (%)		130 (8.7%)	19 (4.8%)	85 (10.9%)	26 (8.1%)	0.002
Peripheral arterial disease, n (%)		114 (7.6%)	26 (6.6%)	58 (7.4%)	30 (9.3%)	0.383
Bleeding history, n (%)		66 (4.4%)	12 (3%)	37 (4.7%)	17 (5.3%)	0.260
COPD, n (%)		165 (11.0%)	54 (13.7%)	80 (10.2%)	31 (9.6%)	0.136
History of stroke/TIA, n (%)		60 (4.0%)	18 (4.6%)	35 (4.5%)	7 (2.2%)	0.130
BMI ≥ 30kg/m^2^, n (%)		301 (20.1%)	75 (19%)	165 (21.1%)	61 (18.9%)	0.596
History of smoking, n (%)	Non-smoker	619 (41.3%)	135 (34.2%)	331 (42.3%)	153 (47.5%)	<0.001
	Former-smoker	568 (37.9%)	152 (38.5%)	309 (39.5%)	107 (33.2%)	
	Active smoker	313 (20.9%)	108 (27.3%)	143 (18.3%)	62 (19.3%)	
Alcohol, n (%)		167 (11.1%)	46 (11.6%)	97 (12.4%)	24 (7.4%)	0.056
History of COVID-19, n (%)		608 (40.5%)	160 (40.5%)	343 (43.8%)	105 (32.6%)	0.003

Categorical variables (%) and continuous variables presented frequently are shown as median and interquartile range. DAPT: Dual antiplatelet therapy; STEMI: ST-elevation myocardial infarction; NSTE-ACS: Non-ST-segment elevation acute coronary syndrome; CCS: Chronic coronary syndromes; CAD: coronary artery disease; CABG: coronary artery bypass graft; COPD: Chronic obstructive pulmonary disease; TIA: transient ischemic attack; BMI: body mass index; COVID-19: Coronavirus disease 2019.

**Table 2:** – Clinical features and concomitant drug treatments of the study patients

		Total n=1500	STEMI n=395	NSTE-ACS n=783	CCS n=322	p value
Systolic blood pressure, mm Hg		130 (120 – 142)	130 (119 – 140)	130 (120 – 145)	130 (119 – 140)	0.003
Diastolic blood pressure, mm Hg		80 (70 – 85)	78 (70 – 85)	80 (71 – 88)	78 (70 – 85)	<0.001
Heart rate, pulse/min		75 (68 – 84)	75 (68 – 85)	77 (69 – 85)	74 (66 – 81)	0.001
PRECISE-DAPT score		15 (8 – 23)	13 (7 – 22)	15 (8 – 24)	15 (8 – 24)	0.128
DAPT score		2 (1 – 3)	2 (1 – 3)	2 (1 – 3)	2 (1 – 3)	<0.001
Hemoglobin, g/dL		13.7 (12.2 – 14.9)	14.0 (12.6 – 15.0)	13.6 (12.1 – 14.8)	13.6 (12.0 – 14.6)	0.005
LDL-cholesterol, mg/dL		110 (85 – 137)	113 (89 – 138)	111 (86 – 138)	106 (77 – 135)	0.030
Left ventricular ejection fraction, n (%)	<%40	152 (10.1%)	53 (13.4%)	82 (10.5%)	17 (5.3%)	<0.001
	%40-49	340 (22.7%)	120 (30.4%)	162 (20.7%)	58 (18%)	
	≥%50	1008 (67.2%)	222 (56.2%)	539 (68.8%)	247 (76.7%)	
CIED, n (%)		24 (1.6%)	3 (0.8%)	19 (2.4%)	2 (0.6%)	0.021
**Concurrent drug treatment**						
Betablocker, n (%)		1310 (87.3%)	337 (85.3%)	688 (87.9%)	285 (88.5%)	0.358
ACEI or ARB, n (%)		1155 (77.0%)	316 (80%)	607 (77.5%)	232 (72%)	0.037
ARNI, n (%)		14 (0.9%)	2 (0.5%)	11 (1.4%)	1 (0.3%)	0.135
SGLT-2 inhibitors, n (%)		138 (9.2%)	42 (10.6%)	62 (7.9%)	34 (10.6%)	0.200
High-moderate intensity statins, n (%)		1244 (82.9%)	326 (82.5%)	650 (83.0%)	268 (83.2%)	0.966
Calcium channel blockers, n (%)		169 (11.3%)	39 (9.9%)	101 (18.3%)	29 (9%)	0.105
Nitrates, n (%)		245 (16.3%)	59 (14.9%)	143 (18.3%)	43 (13.4%)	0.091
Ranolazine, n (%)		225 (15.0%)	51 (12.9%)	132 (16.9%)	42 (13%)	0.109
Trimetazidine, n (%)		265 (17.7%)	60 (15.2%)	145 (18.5%)	60 (18.6%)	0.322
Ivabradine, n (%)		40 (2.7%)	19 (4.8%)	15 (1.9%)	6 (1.9%)	0.015
MRAs, n (%)		163 (10.9%)	61 (15.4%)	84 (10.7%)	18 (5.6%)	<0.001
PPI, n (%)		1174 (78.3%)	303 (76.7%)	607 (77.5%)	264 (82%)	0.179

Categorical variables (%) and continuous variables presented frequently as median and interquartile ranges. DAPT: Dual antiplatelet therapy; LDL: low-density lipoprotein; CIED: cardiac implantable electronic devices; ACEI: angiotensin-converting enzyme inhibitor; ARB: angiotensin receptor blocker; ARNI: angiotensin receptor/neprilysin inhibitor; SGLT-2: sodium-glucose co-transporter-2; MRAs: mineralocorticoid receptor antagonists; PPI: proton pump inhibitor.

**Table 3:** Primary and secondary endpoints

	Total n=1500	STEMI n=395	NSTE-ACS n=783	CCS n=322	p value
**Primary endpoints**					
Hospitalization for any reason, n (%)	193 (12.9%)	53 (13.4%)	108 (13.8%)	32 (9.9%)	0.205
Hospitalization for cardiovascular cause, n (%)	118 (7.9%)	31 (7.8%)	63 (8%)	24 (7.5%)	0.946
Acute myocardial infarction, n (%)	34 (2.3%)	9 (2.3%)	20 (2.6%)	5 (1.6%)	0.573
Stent thrombosis, n (%)	20 (1.3%)	9 (2.3%)	9 (1.2%)	2 (0.6%)	0.127
Target vessel revascularization, n (%)	63 (4.2%)	21 (5.3%)	34 (4.3%)	8 (2.5%)	0.141
BARC type 1 bleeding, n (%)	50 (3.3%)	13 (3.3%)	25 (3.2%)	12 (3.7%)	0.905
BARC type 5, 3, or 2 bleeding, n (%)	8 (0.6%)	1 (0.3%)	5 (0.6%)	2 (0.6%)	0.672
**Secondary endpoints**					
Death for any reason, n (%)	8 (0.5%)	3 (0.8%)	3 (0.4%)	2 (0.6%)	0.684
Death from cardiovascular cause, n (%)	5 (0.3%)	2 (0.5%)	2 (0.3%)	1 (0.3%)	0.777
Stroke, n (%)	5 (0.3%)	2 (0.5%)	3 (0.4%)	0	0.474

Values in parentheses are percentages. BARC: Bleeding Academic Research Consortium.

## Discussion

In patients undergoing medical treatment or PCI for STEMI, NSTE-ACS or CCS, current guidelines have shown that the addition of P2Y12 inhibitors (ticagrelor, prasugrel or clopidogrel) to ASA for appropriate periods has favourable effects on mortality.^3-5^ The combination of ischaemic burden and bleeding risk is crucial in choosing the DAPT and how long it should be continued. Advanced age, presence of ACS, diabetes mellitus, chronic renal failure, angiographic features and high ischaemic risk score (SYNTAX 2, GRACE, TIMI, DAPT) should be considered in the evaluation of ischaemic burden. In the assessment of bleeding risk, history of major bleeding, history of stroke, anaemia, decreased platelet count, malignancy, advanced age, severe liver disease, anticoagulant drug use and high bleeding risk score (ARC-HBR, PRECISE-DAPT) should be considered. DAPT treatment is given by taking into account the recommendations of the current guidelines in a way to balance ischaemic burden, which is an efficacy indicator, and bleeding risk, which is an safety indicator. Fixed- dose combination (ASA 75 mg + clopidogrel 75 mg) treatment was effective and safe in eligible patients with acute or CCSs. In the CURE study, which observed the effects of ASA dose when used alone or in combination with clopidogrel in patients with acute coronary syndrome, higher ASA doses (>100 mg) led to higher bleeding complication rates with lack of additional efficacy.^10^ In atherosclerotic cardiovascular disease, current data recommend a daily ASA dose in the range of 75 to 100 mg. In France, in a study of 380 patients, ASA 75 mg and clopidogrel separately or in fixed-dose combination therapy was shown to improve patient drug compliance, but was not evaluated in terms of efficacy and safety.^6^ Our study is the first prospective, multicentre, observational study to investigate the safety and efficacy of fixed dose combination (ASA 75 mg + clopidogrel 75 mg) DAPT in these patients.

Regarding the primary endpoints, the rate of hospitalization for cardiovascular reasons including heart failure, acute MI, stent thrombosis and rhythm disorders such as atrial fibrillation or ventricular tachycardia was 7.9%. Chronologically, ASA alone, followed by DAPT with ASA and clopidogrel, and finally ASA with ticagroler and/or prasugrel, more potent inhibitors of the adenosine diphosphate receptor P2Y12, have been shown to reduce the risk of myocardial ischaemic events in patients with acute coronary syndrome.^2^ Prospective studies involving patients with acute coronary syndromes or patients undergoing PCI in which clopidogrel therapy was used showed differences in terms of primary endpoints. When we look at the studies conducted on acute coronary syndrome patients, in the TRITON-TIMI 38 study,^11^ MI was found in 9.7% of patients in the group receiving clopidogrel treatment.^11^ In the CLARITY-TIMI 28 study,^12^ the rate of repeat MI in the clopidogrel arm was 2.5%,^12^ and in the CURE study,^13^ the rate of MI was 5.2% in 6,259 patients using clopidogrel.^13^ In the CURRENT-OASIS 7 study,^14^ the MI rate was 2% in the double dose group and 2.6% in the standard dose group,^14^ and in the PLATO study, the MI rate was 6.9% in the clopidogrel arm.^15^ As can be seen, although the MI rate varies in acute coronary syndrome studies using clopidogrel treatment, in our study, this rate was low as 2.3% in the entire population and 2.3% and 2.6% in the STEMI and NSTE-ACS groups, respectively. With respect to studies conducted on CCS patients, in the EXCELLENT study,^16^ MI was observed in 1.8% of 722 patients in the six-month treatment group.^16^ In the PRODIGY study,^17^ MI rate was 4.2% in the short-term DAPT (six months) group, and in CREDO study,^18^ MI occurred in 6.6% of patients in the clopidogrel arm of 1,053 patients.^18^ In a meta-analysis of 10 randomised controlled trials on optimal duration of DAPT after PCI with drug-eluting stents, the MI rate was 1.6% in the short-term treatment group.^19^ Therefore, although the MI rate varies in various studies using clopidogrel treatment, this rate was found to be low as 1.6% in the CCS subgroup.

In a multicentric, prospective study, Oh et al.^20^ investigated the platelet inhibition potential of fixed dose combination of ASA plus clopidogrel and compared with seperate doses in patients who had undergone PCI with drug-eluting stent. The authors demonstrated that the efficacy of platelet inhibition by fixed-dose combination was not different than that of seperate doses of ASA and clopidogrel, with no serious cardiovascular adverse events.^20^ Additionally, in a randomized study, Choi et al.^21^ showed that the pharmacokinetic characteristics of fixed-dose combination of ASA and clopidogrel were bioequivalent to that of seperate administiration of each drug. These pharmacokinetic features may explain the efficacy of fixed-dose combination therapy on clinical endpoints as compared with the administration of the drugs separately.^21^

When we look at stent thrombosis, which is evaluated as another efficacy indicator, different rates have been reported among the studies, mainly due to variations in stent thrombosis definition and patient selection. In the PLATO study,^15^ stent thrombosis occurred in 1.9% in the clopidogrel arm. In the TRITON-TIMI 38 study,^11^ the rate of definite and probable stent thrombosis in the clopidogrel group was 2.4%. In the CURRENT-OASIS 7^14^ study with a short follow-up period of 28 days, the rate of definite stent thrombosis was 0.7% in the double-dose group and 1.3% in the standard-dose group.^14^ Although the stent thrombosis rate varies in ACS studies, in our study this rate was 1.3% in all patients, while it was 2.3% and 1.2% in the STEMI and NSTE-ACS subgroups, respectively. In the EXCELLENT^16^ and PRODIGY^17^ studies, definite and probable stent thrombosis in the six-month DAPT arms were 0.9% and 1.5%, respectively. In a meta-analysis of 10 studies using clopidogrel after drug-eluting stents in CCS patients, the rate of stent thrombosis was found to be 0.52%.^19^ In our study, the rate of definite stent thrombosis was 0.6% in the CCS subgroup. While the rate of urgent TVR was 3.7% in the TRITON-TIMI 38 study,^11^ revascularization after randomization was 11.5% in the CURE study,^13^ any TVR was 13.1% in the CREDO study.^18^ In our study, the TVR rate was 4.2%. These significant differences between the studies may be due to various factors such as the selected stent structure, comorbidities, and follow-up period.

Regarding the secondary endpoint, the rate of stroke was 0.3% in our study. In the CHARISMA study,^22^ the PLATO study,^15^ the CURE study^13^ and the CREDO study,^18^ stroke rates were 1.9%, 1.3%, 1.2% and 0.9%, respectively. We believe such variation is due to different characteristics of the study populations.

Bleeding is used for safety endpoint evaluation of antiplatelet, antiaggregant and anticoagulant therapies. Various bleeding classifications such as The Thrombolysis in Myocardial Infarction (TIMI) bleeding, BARC, REPLACE-2 and GUSTO have been used in studies.^9^ In the CLARITY-TIMI 28 study,^12^ the rates of major bleeding (TIMI) was 1.9% and minor bleeding was 1.3% in the PCI group.^12^ In the TRITON-TIMI 38 study, non-CABG-related TIMI major bleeding was 1.8% in the clopidogrel group.^11^ In the CURRENT-OASIS 7 study,^14^ the rates of CURRENT-defined major and severe bleeding was 2.7% in the double-dose group and 1.9% in the standard-dose group. In the EXCELENT study,^17^ TIMI major bleeding rate was 0.3% in the short-term DAPT group.^16^ In the PRODIGY study,^17^ the rate of BARC type 5, 3, or 2 was 3.5% and of TIMI major bleeding was 0.6% in the six-month DAPT group.^17^ In the CREDO study,^18^ the rate of nonprocedural TIMI major bleeding was 1.2% in the clopidogrel arm. In our study, we used the BARC classification, and the rates of BARC type 1 was 3.3%, while BARC type 5, 3, or 2 was 0.6%. In the STEMI subgroup, BARC type 1 is 3.3%, BARC type 5, 3 or 2 is 0.3%; in the NSTE-ACS subgroup, BARC type 1 is 3.2%, BARC type 5, 3 or 2 is 0.6%, and in the CCS subgroup, BARC type 1 was found to be 3.7%, and BARC types 5, 3 or 2 was found to be 0.6%. It suggests that the differences between the studies may be due to the inclusion and exclusion criteria. Jung et al.^23^ assessed the safety profile of fixed dose combination in a randomized trial. There was no serious adverse events or death in study population.

### Study limitations

The first limitation of our study is the lack of studies comparing other DAPT treatments or ASA and clopidogrel treatments separately. The second is the inclusion of diseases that may pose a high risk for bleeding in the patient exclusion criteria.

## Conclusions

Our study demonstrates that fixed-dose combination DAPT is effective and safe in patients with ACS or CCS, appropriately selected based on their ischaemic burden and bleeding risk.
